# Fæcal Impaction

**Published:** 1901-02-23

**Authors:** 


### FÆCAL IMPACTION.

It is too often the case that the practitioner called upon to deal with cases of dyspepsia, constipation, or diarrhœa, proceeds direct to treatment without undertaking a complete physical examination. Hence many things are missed, and among others, impacted fæcal matter. Dr. Samuel G. Gant,[1] of New York, has recently drawn attention afresh to this subject. He says that these accumulations, causing partial or complete occlusion of the bowel, may be located in any portion of the large intestine. In 60 per cent. of these cases the obstruction will be found in the rectum, in 15 per cent in the sigmoid, in 10 per cent. in the cæcum, and in the rest in other portions of the colon. No age is exempt. In the beginning there is constipation; later there is constipation alternating with diarrhœa, and finally, diarrhœa of the most annoying and persistent character. The general symptoms set up are of the most varied nature, and involve the nervous and the genito-urinary, as well as the digestive systems, while as regards the local changes accompanying the condition, some may be its consequence, some may be its cause. The diagnosis of coprostasis is not always as easy a matter as some people might imagine. It is true that when a hard, large, fæcal mass, uncovered by mucous membrane, is situated in the lower rectum a digital examination quickly reveals its nature. On the other hand, when it is partly covered by the mucosa, or when located in the sigmoid flexure or colon, it is often perplexing to make a positive diagnosis. It must be borne in mind that tumours of the intestine, bladder, vagina, uterus, tubes, ovaries, and prostate sometimes cause intestinal occlusion and a long train of symptoms similar to those caused by coprostasis, and that when the tumour is in the rectum it is often mistaken even by the experienced finger for carcinoma, because the mass pushes the mucous membrane down in front of it, giving to the touch a sensation similar to that of submucous cancer. When the impaction is in the upper regions of the colon it may simulate several other conditions, and indeed it may be impossible to arrive at a diagnosis without an abdominal incision. The impaction in some such cases may be only the result of some other physical condition, such as stricture, tumour or adhesion, for the complete diagnosis as well as for the treatment of which operation may be necessary. In the treatment of fæcal impaction the use of frequent copious enemata is in the first place recommended. In other cases the mass must be broken up, either with the fingers or with a special forceps, by the use of which the enemata are enabled to penetrate the mass; or an anæsthetic may be given, the anus stretched, and the mass removed either whole or in sections. When these masses are situated higher up the same enemata may be used, and when the tumours come down into the rectum they may in turn be broken up; and lastly in some cases an operation may be required, a permanent artificial anus being established when the impaction has been due to stricture or tumour. The enema recommended for dislodging impacted fæces consists of castor oil one ounce, glycerine two ounces, and soap suds a pint.

The Post-Graduate, January, 1901.

